# Unusual Location of Primary Hydatid Cyst: Soft Tissue Mass in the Supraclavicular Region of the Neck

**DOI:** 10.1155/2012/484638

**Published:** 2012-08-29

**Authors:** Slim Jarboui, Abdelwaheb Hlel, Alifa Daghfous, Mohamed Ali Bakkey, Imed Sboui

**Affiliations:** ^1^Department of General Surgery, Sidi Bouzid Hospital, 9132 Sidi Bouzid, Tunisia; ^2^Department of Radiology, Trauma Center of Ben Arous, 2013 Ben Arous, Tunisia

## Abstract

Cystic hydatid disease is a zoonosis caused by *Echinococcus granulosus*. It may affect any organ and tissue in the body, in particular the liver and Lung. Musculoskeletal or soft tissue hydatidosis accounts for about 0,5%–5% of all echinococcal infections in endemic areas and is almost secondary to the hepatic or pulmonary disease (Karaman et al., 2011; Dirican et al., 2008; Kouskos et al., 2007). *Case Presentation*. We report an unusual case of primary subcutaneous hydatidosis in the left supraclavicular region of the neck. A 53-year-old female patient was admitted with three-month history of pain and gradually growing mass located in the left supraclavicular region. Physical examination revealed a moderately hard, painful, and erythematous mass. The blood cell count was normal. Computed tomography demonstrated, a multilocular cystic lesion with thin borders and thin wall. The mass is binocular and extends to the scapula. CT showed no involvement of the lung. From these signs, the patient was diagnosed as having abscess (bacterial infection or tuberculosis). The diagnosis of *Echinococcus granulosus* infection was made per operatively after visualization of the cyst wall and the daughter cysts. Following irrigation of cystic cavity with hypertonic saline solution, the cyst wall was excised along with a portion of surrounding tissue. Histopathological examination of the specimen confirmed the hydatid origin. Hemagglutination tests for *Echinococcus* and ELISA were negative. Ultrasound of the abdomen was normal. The patient received albendazole (400 mg/day) for 8 weeks postoperatively. No sign of recurrence could be detected by physical examination and imaging (CT) at 4-month followup. *Conclusion*. The case illustrates that echinococcal disease should be considered in the differential diagnosis of every cystic mass in every anatomic location, especially when it occurs in endemic areas.

## 1. Introduction

Hydatid disease is an important infestation caused by the parasite *Echinococcus granulosus* and still common in countries in the temperate zones, including the Mediterranean countries, the Middle East, South America, New Zealand, Australia, and Southeast Asia and China [1, 2]. Hydatids are mostly found in the liver and/or in lungs for physiopathological reasons, but several other encysting sites are possible; bone is included. The musculoskeletal involvement has been registered in only 1–4% of the cases [2, 3]. It has been hypothesized that the presence of lactic acid in the muscle does not allow the larvae to grow into cysts. Only few cases of primary subcutaneous hydatidosis have been reported, and even in regions with endemic *Echinococcosis*, hydatidosis of cervicofacial region is extremely rare.

We would like to point out that, regardless of the site involved, this zoonotic infection should always be considered in the differential diagnosis of any cystic lesion especially in endemic areas.

## 2. Case Report

A 53-year-old woman presented to our hospital with a three-month history of painful mass growing on the left lateral side of the neck. She reported neither fever nor a weight loss. Upon physical examination, some hard, painful, and fluctuant mass of 10 × 7 cm was palpable on the supraclavicular region of the lateral neck. The overlying skin was inflammatory. We did not find any regional lymphadenopathy. The blood cell count was normal except an increased erythrocyte sedimentation rate (ESR 40 mm/h). No abnormality was found in chest X-ray.

**Figure 1:**
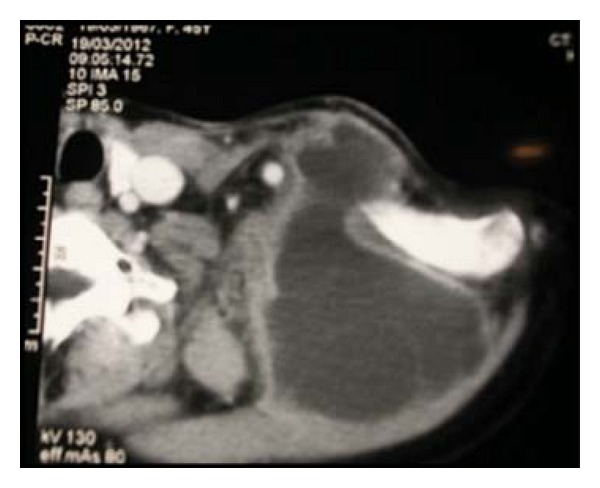
CT scan demonstrated cystic structure in the left supraclavicular region with multivesicular lesions.

**Figure 2:**
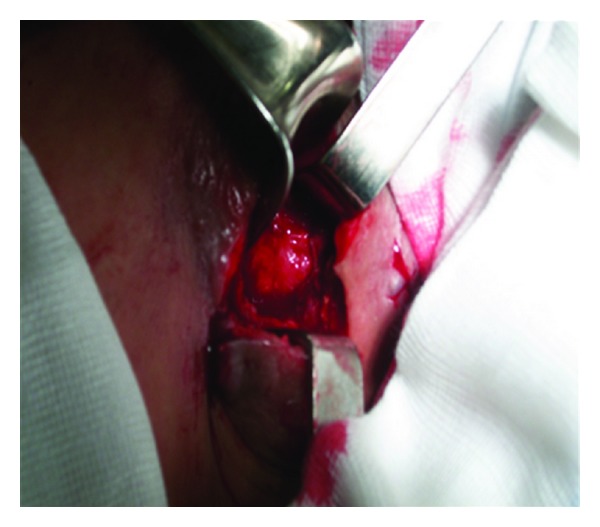
Subcutaneous hydatid cyst in the left supraclavicular region of the neck.

**Figure 3:**
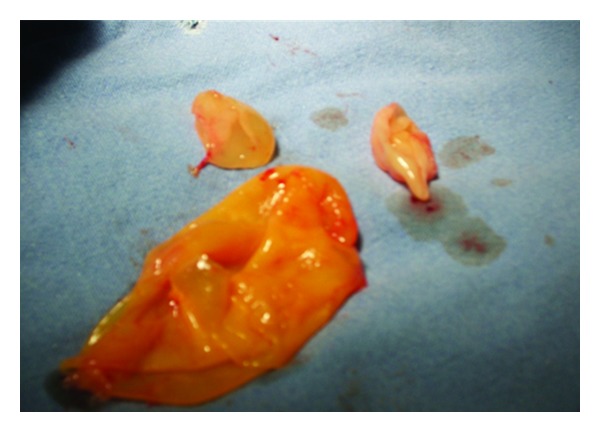
A view of the resected cyst and its germinative membrane with daughter cysts.

**Figure 4:**
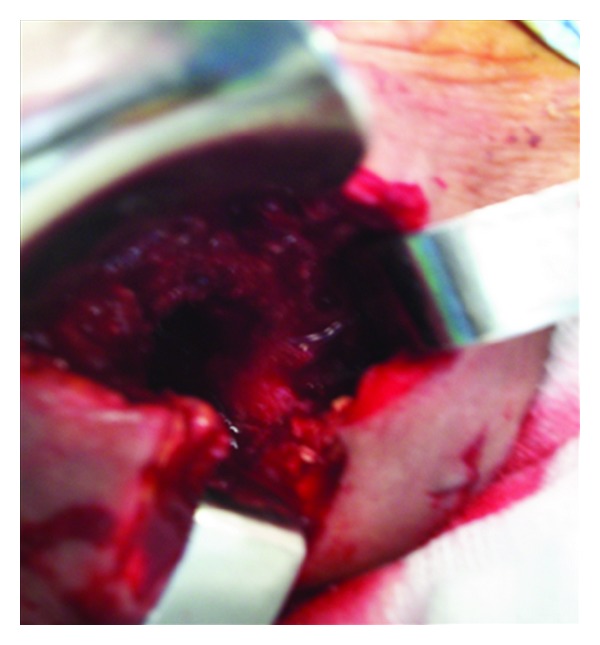
Operative field after cyst resection. The cyst pouch was irrigated with protoscolicidal solutions.

CT scan demonstrated a binocular mass on the left supraclavicular region with cystic aspect. Its dimensions are 14 × 10 cm, and it contains numerous cystic formations with thin outlines (Figure 1). From this clinical and radiographic signs, the patient was diagnosed as having pyogenic abscess or tuberculosis, and its excision or drainage was planned. The diagnosis of *Echinococcus granulosus* infection was confirmed perioperatively after visualization of the cyst wall and daughter cysts (Figures 2 and 3). Following irrigation of cystic cavity with hypertonic saline solution, the cyst wall was excised with a portion of overlying skin and muscle (Figure 4). Histopathological examination of the specimen revealed hydatid cyst and showed an inflammatory tissue reaction surrounding “parasite-like cyst.” Scoleces were also detected within the surgical specimen. The patient was subjected to ultrasound of the abdomen and urologic examination. Hemagglutination tests for *Echinococcus* and ELISA were negative. The investigation did not reveal any extracervical sites for other hydatid cyst. The patient received Albendazole (400 mg/day) for eight weeks postoperatively. No sign of recurrence could be detected by physical examination and imaging at the 4-month followup.

## 3. Discussion

Hydatid cyst disease is still a major health problem in agricultural countries including Tunisia. The parasite is named *Echinococcus granulosus*, and humans can be an incidental intermediary host in the life cycle of the parasite. Although cysts are most commonly located in the liver (50–65%) and the lungs (20–30%), multiorgan involvement is seen in 20–30% of cases [1–4]. The musculoskeletal involvement has been registered in only 1–5%. It has been hypothesized that the presence of lactic acid in the muscle does not allow the larvae to grow into cysts [2, 4]. Soft tissue hydatid cysts occur in 2-3% of cases reported from endemic areas [1, 3, 4].

Diagnosis of *Echinococcus* should be considered when slowing growing soft tissue is present in patient from a rural area especially endemic country. The clinical manifestation of the disease is a result of the localization and pressure effect of the slow-growing cyst on the infected organ. The main complaints of patients have been pain and sense of malaise [1, 4–6]. As in our case, clinical findings and physical examination did not reveal the correct diagnosis. Our initial diagnosis was soft tissue suppuration or tuberculosis. *Echinococcus granulosus* infection may also mimic malignancy because of sudden enlargement of the cyst and the formation of structural deformities in the tissue over time.

*Ecchinococcosis* is diagnosed essentially by the patient's history, physical examination findings, radiologic imaging modalities, and serological tests. Although imaging methods usually exhibit nonspecific findings, they may be helpful for making the diagnosis. Ultrasonography, computed tomography, and magnetic resonance imaging can show the association of the cyst with neighboring tissues, the internal laminar wall of the cyst, and intraluminal daughter cysts [1, 5–7]. CT and US show the hydatid sands in purely cystic lesion as well as floating membranes, daughter cysts, and vesicles most clearly are the methods of choice for searching for the pathognomonic criteria of hydatid cyst [1, 2]. For Mekki et al., MRI appears to be the most useful imaging technique when a complex or solid pattern is present [8]. Our patient has a CT of the neck and chest when he was admitted into our clinic, so we did not need any other radiologic imaging. Although imaging techniques are reported to enlighten the diagnosis of hydatid cyst, the diagnosis was missed in the CT scan of the neck and chest in our case, and the mass was reported as a simple cystic structure. Abdominal and chest X-rays, ultrasound, and CT scans should be performed in order to investigate various organ involvements, particularly liver and lungs. Eroglu et al. and Iynen et al. have reported an unusual case of hydatid cyst found in the neck, and just like our paper, there was no pulmonary or hepatic involvement [2, 9].

To confirm the diagnosis, serologic tests, including direct hemagglutination, latex agglutination, immunoelectrophoresis, skin tests, and enzyme-linked immunosorbent assay (ELISA), are widely used [1, 4, 5, 7]. The sensitivity of various serological tests used for hydatid disease varies from 64 to 87% [4]. However, all serologic tests have low diagnostic sensitivity and specificity. Hydatid serology is only valuable when it is positive, and negative serologic test does not exclude the diagnosis. We performed hemagglutination test and ELISA test in our patient, and the result was negative [1–3, 7–11]. So, the diagnosis of *E. granulosis* in our patient was made on the basis of the exposure in an endemic area like Tunisia, the CT imagings, the operative findings, and on histopathological examinations. Serological tests were negative, and specific tests like Western Blot assay for specific *E. granulosis* proteins and enzyme immunoassay for specific IgG were not made.

The best treatment option is total surgical excision without opening the cyst. If the cyst cannot be excised without opening, the fluid contents should be removed, the laminated membrane should be totally excised, and the cyst pouch should be irrigated with protoscolicidal solutions [2, 3, 7, 8, 11]. This approach was made in our patient, and the cyst cavity was excised partially because of the deep location of the cyst and its vascular relationships. Subcutaneous located cysts are more prone to rupture since they have not been diagnosed preoperatively. Medical treatment with antihelmintic drugs, such as mebendazole and albendazole, should be included especially for disseminated, inaccessible hydatidosis and for patients who do not favor the morbidity of an operative process [2, 9, 11–15]. These drugs may also play an important role in conjunction with surgery, both preoperatively for sterilization of the cyst and postoperatively in case of spillage [2, 13, 15].

## 4. Conclusion

This case illustrates that echinococcal disease should be considered in the differential diagnosis of every cystic mass in any anatomic location, especially when it occurs in areas where the disease is endemic like Tunisia.
